# Simian Foamy Viruses in Central and South America: A New World of Discovery

**DOI:** 10.3390/v11100967

**Published:** 2019-10-20

**Authors:** André F. Santos, Liliane T. F. Cavalcante, Cláudia P. Muniz, William M. Switzer, Marcelo A. Soares

**Affiliations:** 1 Departamento de Genética, Universidade Federal do Rio de Janeiro, Rio de Janeiro 21941-617, RJ, Brazil; andre20@globo.com (A.F.S.); liliane.tavaresdefaria@gmail.com (L.T.F.C.); claudia.muniz16@gmail.com (C.P.M.); 2 Laboratory Branch, Division of HIV/AIDS Prevention, National Center for HIV/AIDS, Hepatitis, STD, and TB Prevention, Centers for Disease Control and Prevention, Atlanta, GA 30329, USA; bis3@cdc.gov; 3 Programa de Oncovirologia, Instituto Nacional de Câncer, Riod e Janeiro, RJ 20231-050, RJ, Brazil

**Keywords:** spumaretrovirus, new world primates, simian retrovirus

## Abstract

Foamy viruses (FVs) are the only exogenous retrovirus to date known to infect neotropical primates (NPs). In the last decade, an increasing number of strains have been completely or partially sequenced, and molecular evolution analyses have identified an ancient co-speciation with their hosts. In this review, the improvement of diagnostic techniques that allowed the determination of a more accurate prevalence of simian FVs (SFVs) in captive and free-living NPs is discussed. Determination of DNA viral load in American primates indicates that oral tissues are the viral replicative site and that buccal swab collection can be an alternative to diagnose SFV infection in NPs. Finally, the transmission potential of NP SFVs to primate workers in zoos and primate centers of the Americas is examined.

## 1. Introduction

Spumaretroviruses are complex, exogenous retroviruses in the *Spumaretrovirinae* subfamily known to infect different mammalian orders, such as nonhuman primates (NHPs), felines, bovines and equines [1]. In NHPs, spumaretroviruses are also called simian foamy viruses (SFVs). Despite being the only reported exogenous retrovirus known to infect neotropical primates (NPs), as first reported in 1973 [2], little is known about this viral infection. Recently published studies using improved molecular and serologic techniques for SFV diagnosis in NPs have shed light on the prevalence, transmission routes and zoonotic potential of these NP viruses.

## 2. Neotropical Primates: Taxonomy and Evolution

**Figure 1:**
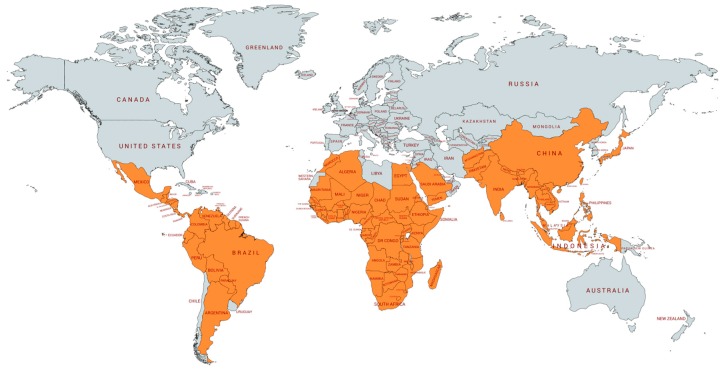
Global primate distribution. In orange, countries with native species of primates. Data were extracted from IUCN/SSC Primate Specialist group web site www.primate-sg.org/threat_primate_habitat_country/ on August 15th. Graph art was generated using mapchart.net.

The word “primate” is derived from Latin *primat* that means prime or first rank. The Primates order has the third most abundant number of species among mammals, only behind Chiroptera (bats) and Rodentia (rodents) [3]. Although the exact number of species is still in discussion with constant changes in taxonomic classification, there are between 261 and 504 species described to date divided into 16 families and 79 genera [3,4]. Primates are distributed across four global regions: Latin America, mainland Africa, Madagascar, and Asia, covering 90 countries (Figure 1) [3]. Common features of the Primates order include a large brain in relation to the body size, accurate binocular color vision, opposable thumbs and a sophisticated social system. The common ancestor of the Primates order is estimated to have originated about 60–80 million years ago (MYA) based on evidence of small mammals adapted to live in trees and with the oldest fossil found in Africa [5].

Primates radiated to five infraorders, of which the infraorder Simiiformes emerged about 36–50 MYA and is divided in the parvorders Catarrhini (Old World monkeys, great apes, gibbons, and humans) and Platyrrhini (neotropical monkeys). The parvorder Catarrhini consists of three families: Cercopithecidae, Hominidae and Hylobatidae. The Cercopithecidae family, also known as Old World primates (OWPs), is the largest family, with 32 genera and 138 species described living in Africa and Asia [4]. Examples of OWPs include the *Macaca* (macaques), *Papio* (baboons), *Cercocebus* (mangabeys) and *Mandrillus* (mandrills) genera, all primates lacking prehensile tails. The Hylobatidae family harbors Asian primates known as gibbons, considered small apes [6]. The Hylobatidae is considered a sister clade of the Hominidae family, composed of the great apes (the largest primate species) and includes four genera: *Pongo* (orangutan), *Gorilla* (gorilla), *Pan* (bonobo and chimpanzee) and *Homo* (human) [4].

The parvorder Platyrrhini, also known as neotropical primates (NPs), is composed of Latin American primates descendent from African Cercopithecidae primates that reached South America about 40 MYA [4,7]. The spread of NPs in South and Central America resulted in a broad radiation that permitted the occupation of a large range of biomes from Mexico to the Argentinian Patagonia, leading to a great diversity of morphology and body size [8]. NPs are small to mid-sized animals, ranging from the world’s smallest primate pigmy marmoset (*Cebuella pygmae*; 14–16 cm in length) to the Southern muriqui (*Brachyteles acrachnoides*; 55–70 cm in length). Other unique features of Platyrrhini include a flat nose compared to OWPs (originating the name of the parvorder) and a prehensile tail. NPs also lack trichromatic vision, which is characteristic of OWPs [9]. In contrast to OWPs, most NP species constitute monogamous pairs, and provide extensive paternal care of young [10]. With respect to diet, NPs eat fruits, nuts, flowers, insects, bird eggs, spiders, and small mammals [11].

Platyrrhini is divided into three families (Atelidae, Cebidae, and Pitheciidae), 21 genera and at least 170 species according to recent molecular analyses [4], of which 42% are threatened (www.primate-sg.org/primate_diversity_by_region/). Since the 2000s, 19 novel species and subspecies have been described in the region, with the most recent being a new titi monkey species (*Plecturocebus grovesi* sp. nov.), described in 2019 [12].

## 3. Diversity and Origin of SFVs in the Americas

SFVs have been shown to naturally infect most nonhuman primates (NHPs), including NPs, OWPs, and prosimians [13,14]. For over 60 years of spumavirus study, most research focused on SFVs in OWPs. In 1973, the presence of a syncytium-forming virus was first detected in a spider monkey (*Ateles* sp.) brain culture, classified then as SFV-8 [2] and currently named SFVaxx after the revision of foamy virus nomenclature in 2018 [15]. The original classification using numbers was based on serologic neutralizing activity, with consecutive numbers used for those isolates with undetectable or weak neutralizing activity to known SFVs indicative of infection with a divergent variant. The current SFV classification uses a three-letter code for the host species name with the first letter of the host genus and the next two letters derived from the first two letters of the species or subspecies. If the species or subspecies is unknown, the letters “xx” are used. Hence, SFVaxx refers to SFV from an *Ateles* monkey for which the species is not known. In 1975, an SFV infecting capuchin monkeys (*Cebus* sp.) was isolated and called SFV-9 [16]. In 1976, another strain of SFV was isolated from red uacari (*Cacajao rubicundus*) lymphocytes in a co-culture with kidney cells from a nocturnal monkey (*Aotus* sp.) [17]. Early in the 1980s, a fourth neotropical SFV was characterized in skin explants of 46 healthy white-tufted marmosets (*Callithrix jaccus*) [18]. Not until 2007 was the complete genome of SFVaxx obtained, 34 years after it was first isolated [19]. In 2010, complete SFVssc and SFVcja genomes, which infect squirrel monkeys (*Saimiri sciureus*) and white-tufted marmosets (*Callithrix jaccus*), respectively, were reported [20]. 

While phylogenetic analysis of short polymerase (*pol*) sequences demonstrated the co-evolution of SFVs with their NHP hosts [14], only one sequence from SFVaxx was available at the time to fully understand the evolutionary trajectory of SFVs in NPs. Phylogenetic analysis of the three complete SFV genomes from NPs with those from OWPs and a prosimian showed SFVs clustering into three major clades, reflecting the evolutionary split between NP (parvorder Platyrhini), OWP (parvorder Catarrhini), and prosimian (Strepsirrhini suborder) hosts [20]. More recently, complete SFV genomes were obtained from *Sapajus xanthosternos*, the yellow-breasted capuchin (SFVsxa), and *Brachyteles arachnoides*, the wooly spider monkey (SFVbar) [21,22]. Additionally, partial SFV *pol* and/or LTR/*gag* sequences (around 500-bp) were obtained from SFV strains infecting 20 different NP species from 10 genera, encompassing all three NP families (Table 1) [23,24,25]. Nonetheless, the complete or partial characterization of these few strains is still poor when compared to the wide diversity of NPs, with more than 150 species described. Furthermore, another 16 species from 11 NP genera had indirect evidence of foamy virus (FV) infection, characterized by diagnostic PCR, Western blot detection and/or detectable DNA viral load (VL) by quantitative PCR (qPCR) (Table 1). The size of the DNA fragments generated by these PCR techniques (192-bp) was, however, too small to enable robust phylogenetic inferences from those species, since this region is very conserved among the different strains and therefore has a low phylogenetic signal for resolution of related strains. 

**Figure 2:**
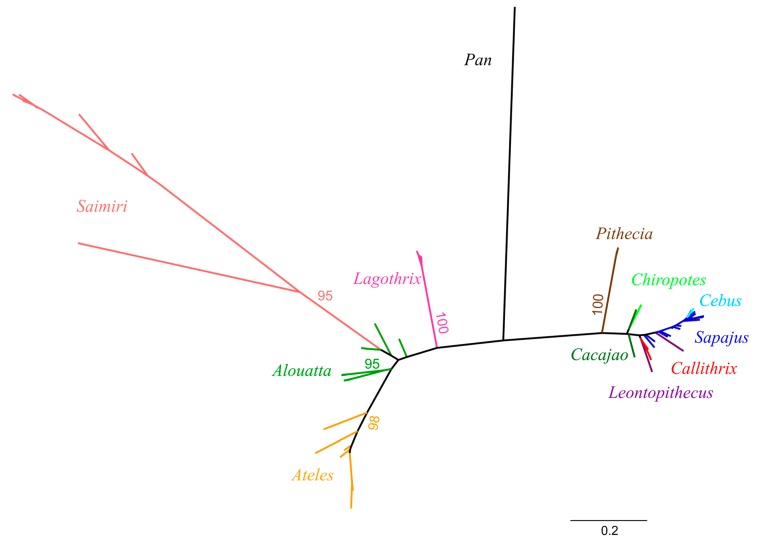
Phylogenetic relationships of simian foamy viruses from neotropical primates (NPs). Unrooted tree inferred by maximum likelihood analysis of an alignment of 411 nucleotides of partial polymerase sequences with 1000 bootstrap replicates. Different NP genera and bootstrap values are indicated by distinct colors. Distance bar is shown at the bottom.

Phylogenetic analysis of the genetic sequences of NP SFV isolates has allowed for the determination of their evolutionary history, which shows distinct evolutionary lineages. Furthermore, genetic characterization of endogenous FVs integrated into mammals, reptiles, amphibians and fish [14,26,27,28,29,30,31] has permitted elucidation of the genetic relationships between exogenous and endogenous FVs, revealing strong evidence of an ancient co-speciation of FVs with their hosts since the origin of marine vertebrates around 500 MYA [27]. The co-speciation hypothesis of SFVs with their primate hosts has also been demonstrated in OWPs and NPs at the family, genus and species levels occurring around 43 MYA [24,25,32]. The phylogeny of NP SFVs also reflects the evolutionary relationships of their hosts, but with some exceptions. For example, SFVs from monkeys in the Pithecidae family (Pithecia, Cacajao, Chiropotes) are not monophyletic as expected with co-evolution and SFV *pol* sequences from Saimiri are paraphyletic to those from monkeys in the Cebidae family instead of being sister taxa in accordance with the co-evolutionary hypothesis (Figure 2). These unexpected phylogenetic relationships may result from only short sequences being analyzed or from cross-species infections, as further discussed below.

**Figure 3:**
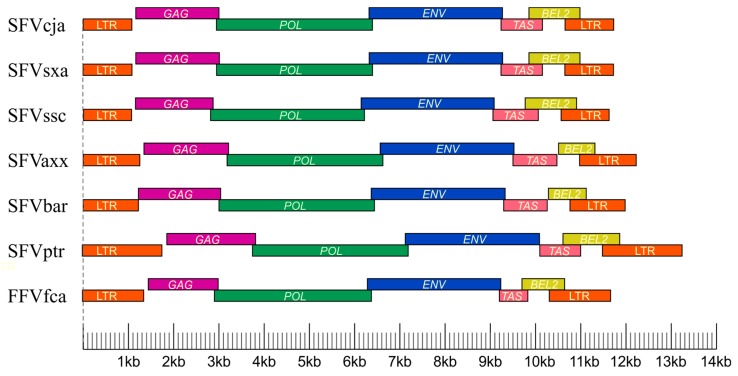
Structural organization of complete simian foamy virus (SFV) and feline foamy virus (FFV) genomes. SFVcja (*Callithrix jaccus*), SFVsxa (*Sapajus xanthosternos*), SFVssc (*Saimiri sciureus*), SFVaxx (*Ateles* sp.), SFVbar (*Brachyteles aracnoides*), SFVptr (*Pan troglodytes*), FFVfca (*Feline catus*). LTR, long terminal repeat; GAG, group specific antigen, POL, polymerase; ENV, envelope; TAS, transcriptional activator; BEL2, between the ENV and LTR.

Comparison of complete SFV genomes among primate hosts revealed some interesting features (Figure 3). For example, the mean size of the *pol* gene is highly conserved among SFVs infecting OWPs and NPs (average of 3435-bp and 3436-bp, respectively) [21]. Similarly, the envelope (*env*) gene lengths are also comparable with a mean size of 2949-bp in NP SFVs and 2962-bp in OWP SFVs. The length of these two genes also appears to be conserved in feline and equine FVs. The exception is the bovine FV *pol* gene, which is 3660-bp in length [21]. The size of the *gag* gene coding for the group specific antigen is conserved within both NP (range of 1817–2071-bp) and OWP SFVs (range of 1872–1974-bp) [21,22,33]. The exception is SFVssc, which has the shortest *gag* gene (1716-bp) of all SFVs described to date. The long terminal repeat (LTR) region has a mean length of 1696-bp among OWP SFVs, with the longest LTR in SFVs infecting a pileated gibbon (SFVhpi), at 2074-bp [21,33], while an SFV infecting Cebidae has the shortest LTR among FVs (1061–1080-bp). SFVaxx has LTRs ranging from 1129-bp (SFVbar) to 1251-bp (SFVasp) in length [21,22]. Whether gene or LTR length differences among SFVs affect their biological functions remains to be elucidated.

## 4. NP SFV Prevalence and Viral Detection Methodologies

Very little is known about the prevalence of SFV in wild NP populations, while studies of captive animals are relatively common (Table 2). A seminal study in 1975 with *Ateles sp.* (spider monkeys) determined that 61% of specimens had antibodies against SFVaxx [16]. Another study conducted in the 1980s with 90 marmosets (*Callithrix jaccus* and *Saguinus* sp.) living in a colony in the United States (U.S.), found a 54% SFV seroprevalence only in *Callithrix* specimens [18]. However, these studies were performed using in-house serological assays that were not validated and standardized, and therefore the reported SFV serological prevalence may have been overestimated by the lack of specificity of the test and/or possible cross-reactivity.

Decades later in 2013, a large study examined 332 NP samples from 15 genera using molecular testing to detect 192-bp *pol* NP SFV sequences in peripheral blood mononuclear cells (PBMCs), including—for the first time—samples collected from wild monkeys [23]. The PCR assay used in this study had a 100% sensitivity using PBMC specimens from Western blot (WB)-positive monkeys from seven NP genera (*Alouatta*, *Aotus*, *Ateles*, *Cacajao*, *Callithrix*, *Cebus*, and *Pithecia*), except for *Saimiri* specimens, with four PCR-positive specimens being WB-negative [34]. The WB used combined antigens from extracts of CfTh2 cells infected with two NP SFV isolates from *Callithrix jaccus* (SFVcja) and *Ateles* sp. (SFVaxx), representing two out of the three NP families, and had shown sensitivity and specificity both > 94% using > 100 SFV+ and SFV– NP specimens [25]. Muniz et al. showed that SFV prevalence among captive specimens was 23% (61/267) versus 29% (19/65) in wild NPs [23]. The molecular prevalence found in this study is lower than that described for African OWP SFVs in wild monkeys (60–100%), including mandrills (*Mandrillus sphinx*), red colobus monkeys (*Piliocolobus badius badius*), baboons (*Papio cynocephalus*) and chimpanzees (*Pan troglodytes*) [35,36,37,38]. In comparison, the molecular prevalence of SFV in captive Asian macaques is 39% among rhesus macaque (*Macaca mulata*) [39], while a seroprevalence of 64–94% has been reported in captive cynomolgus macaque (*Macaca fascicularis*) [40]. The lower SFV prevalence in NPs may reflect a high number of specimens from juveniles, since SFV seroprevalence has been shown to be associated with age. For instance, in a study of a colony of tonkean macaques (*Macaca tonkeana*), SFV seroprevalence was shown to be lower in infants (5%), intermediate in sub-adults (44%) and high in adults (89%) [41]. The same age and seroprevalence relationship has been observed in baboons and cynomolgus macaques [1,40,42]. However, no age of specimens was available in Ref. [23], since that was a study conducted with retrospective samples.

In 2015, a new study estimated the SFV prevalence among Peruvian primates in zoos, rescue centers and the illegal trade market using both molecular and serological assays [25]. The seroprevalence of SFV in NPs from Peruvian zoos using both enzyme immunoassay (EIA) and Western blot (WB) was higher (47%) than those in rescue centers of free-living primates (19%; *p* = 0.001) and similar to those reported in NPs from U.S. zoos (45%). Noteworthy was the SFV prevalence of 43% in Peruvian zoos and 22% in rescue centers in *Lagothrix lagothicha* (wooly monkey), indicating an increased SFV infection in captivity. The lower prevalence in rescue centers could reflect testing of younger animals. Overall, the WB assay in that study showed a total prevalence of 78%, higher than those of the diagnostic PCR assay (19%) and for a PCR test that detected a 495-bp *pol* sequence (26%), indicating that WB more accurately detects previous or current SFV infection. The low efficiency of the diagnostic PCR method could be explained by the high genetic variability of NP SFVs. The *pol* genetic diversity between strains of SFV infecting genera within the same primate family is around 12% in the Cebidae and 31% in the Atelidae families. In contrast, a higher *pol* divergence (41%) occurs between SFV from the Atelidae and Cebidae [23]. The lower sensitivity of the PCR assay could also be explained by the low DNA proviral load (pVL) of NP SFVs in blood cells, with an average of 800 SFV copies/10^6^ cells reported [43,44], very similar to that found in OWPs [45,46]. Furthermore, these findings suggest that SFV does not expand to significant levels in this compartment [46,47].

Another study in 2015 compared the serological and molecular prevalence of NP SFV at the Rio de Janeiro Zoo and the Center of Primatology of Rio de Janeiro state, confirming that WB is more sensitive for detecting SFV infection in NPs [24]. However, as some WB-seronegative animals in the study were PCR-positive, the use of both techniques for SFV detection is needed for epidemiologic studies. Monkeys testing positive with either or both tests would then be considered SFV-positive. In this study, the NP SFV prevalence was similar to that found in Peru, with a WB prevalence of 43% and a molecular prevalence of 29% in captive animals. Of the 140 specimens analyzed, 51% were positive in at least one test. Although serology in these recent studies was shown to be more sensitive for SFV detection, preparation of large amounts of antigens used in the serologic assays may be costly and is dependent on the laboratory having biosafety facilities and equipment for cultivating FV strains in cell culture [24,25]. Good alternatives to that limitation include cloning of SFV *gag* genes and expressing them in bacterial systems, or the use of synthesized peptides from *gag* gene sequences to detect antibodies, which have been used in feline foamy virus (FFV) and SFV research [48,49]. More recently, a new study developed a real-time PCR (qPCR) methodology to diagnose and measure SFV DNA pVL in NPs with a high sensitivity and specificity [43]. Of animals with detectable SFV DNA pVL, 90–96% were also WB-positive. NP SFV prevalence using the different methodologies is summarized in Table 2.

An important concern for detecting SFV in the blood of NPs is the small size of these animals, which limits the amount of blood collected. Consequently, there is typically only low amounts of genomic DNA available to be used for diagnostic PCR [23,24]. Considering this, qPCR can be used as a simpler alternative to SFV WB detection with the same sensitivity but using less DNA. Another alternative is the use of buccal swabs for PCR detection of NP SFVs. In addition to being a less invasive collection method, SFV replication is higher in the oral cavity of OWPs [45,50], which should increase the sensitivity of detecting SFV infection by using molecular methods [51,52]. For NPs, the SFV DNA pVL found in oral tissues ranges between 20–142 million copies/10^6^ cells [43], which is higher than that reported for SFV-infected rhesus macaques (*Macaca mulatta*) using salivary gland, tonsil and tongue specimens (500–100,000 SFV copies/10^6^ cells) [46]. A recent study showed that the DNA pVL found in oral tissues of NPs with matching PBMC samples was up to 8000 copies/10^6^ cells higher than those observed in blood cells, showing that oral tissues are better specimens for SFV detection [43]. The authors of that NP study also showed that the DNA pVL in PBMC is a proxy for the detection of proviral DNA in PBMC by nested PCR with qPCR again being more sensitive. However, only 45% of NPs in that study had SFV detected in oral tissues, which could underestimate the true prevalence in NPs if only buccal swabs are used for diagnosis [43]. 

## 5. NP SFV Epidemiology and Transmission

In OWPs, SFV proviral DNA has been found in many tissues and cell types, including in oral-respiratory tissues and peripheral blood cells [47,53]. Nonetheless, viral expression and replication appear to be restricted to the oral mucosa, as studies have shown high levels of viral RNA only at this site [1,47]. It has also been reported that oral mucosa epithelial cells act as a virus reservoir [50]. Thus, scratches, bites and grooming constitute the main routes of horizontal OWP SFV transmission, mainly via parental care and aggressive behavior in territorial and sexual partner disputes [1]. 

For NPs, SFVs have been molecularly detected in liver specimens [23], oral tissues [43] and peripheral blood cells [23,24,25,43]. The SFV DNA proviral load (pVL) in oral mucosa is similar for NPs [43,44] and OWPs [45,46], indicating this compartment as a virus reservoir for FV infecting primates in general. The higher SFV DNA pVL found in oral tissues of SFV-infected NPs is compatible with horizontal transmission via contact with saliva in biting or grooming, as described for OWPs [1,50]. However, studies in NPs have not yet been conducted to determine SFV RNA levels in these compartments and their correlation with transmission. Since SFV isolates have been obtained from oral swabs of NPs, it would not be surprising that comparable SFV RNA levels are found in NPs as in OWPs [45].

Murray et al. proposed a model in which SFV primary infection occurs in blood and migratory cells, such as macrophages or leukocytes, which carry the virus to the basal epithelium of oropharyngeal tissues, with subsequent FV replication in differentiated epithelial cells [50]. A cynomolgus macaque (*Macaca fascicularis*) that was infected by SFV after a controlled blood transfusion from an SFV-infected donor macaque showed detectable DNA VLs in saliva 29 weeks after infection and detectable RNA VLs after 39 weeks [54]. In this scenario, it may take months for a newly infected animal to become capable of transmitting SFV. This model could explain why only 45% of SFV-infected NPs had DNA VL detectable in oral tissues [43]. Similar results were observed in free-ranging rhesus macaques (*Macaca mulatta*), of which about 30% of SFV-positive animals did not have detectable RNA VLs in saliva [45]. The RNA VLs in this study also correlated with the age of the macaques, with older monkeys having higher SFV RNA levels [45].

SFVs can also be transmitted vertically but studies have been limited to small numbers of species. Blasse et al. showed that mother-to-offspring transmission of SFV is frequent among Western chimpanzees (*Pan troglodytes verus*) [55]. However, vertical transmission was rare in a study of captive *Macaca tonkeana* [41]. Even less is known about vertical transmission in NPs. A five month-old *Chiropotes* infant in quarantine was SFV-positive by both WB and *pol*-integrase PCR testing, indicating a possible vertical transmission [24]. However, samples from the mother were not available for testing to confirm this hypothesis, as the infant was from a rescue center. Studies of specimens collected in utero, perinatally, and from breast milk are needed to better understand the risks of vertical transmission of SFV.

Sexual transmission occurs for animals infected with other retroviruses, like primate lentivirus (SIV), but is rare [56,57]. However, little is known about the potential for sexual transmission for SFV. Differences in the NP SFV prevalence in males and females have not been observed, suggesting that if sexual transmission occurs it is equally likely from male to female and vice versa [23,24,25]. Differences in the SFV prevalence among different age groups of NPs have been reported, suggesting that horizontal transmission is likely more common than vertical transmission. Ghersi et al. found that the SFV prevalence increased with age among captive NPs in Peru, with 0% in infants, 30–50% in juveniles, 50–58% in sub-adults and 55–64% in adults [25]. In NPs from Peruvian rescue centers in that study, the SFV seroprevalence was higher in adults (32%) than in juveniles (17%). These findings are congruent with the SFV prevalence found in different age groups of OWPs [1,40,41]. However, Muniz et al. did not find a significant difference in SFV prevalence between juveniles (73%) and adults (87%) in vivaria in Brazil [24]. This SFV prevalence disparity may reflect a more confined environment of the vivaria in Brazil, which can increase the stress of the animals and, consequently, viral transmission. Another explanation could be different transmission rates by primate species. For example, when SFV results of *Sapajus* were analyzed separately, as they represented one third of the samples in that study, infected NPs were on average 6.8 years older than the uninfected NPs [24]. Additional studies with NPs from other countries, environments, and with more species will help to better define NP SFV prevalence by age. Nonetheless, the finding of high VLs in the oral mucosa and in older animals, and the increased SFV prevalence in older monkeys, suggests that horizontal transmission likely occurs via aggressive behaviors, such as biting, as monkeys become adults [1].

Similar to SFV-infected OWPs, evidence of pathogenicity has not been reported in SFV-infected NPs, though systematic studies have not been conducted for both parvorders. In rhesus macaques, SIV and SFV co-infection was reported to accelerate immunodeficiency related death (75% of deaths in 39 weeks) in comparison to those infected with only SIV (37% of deaths in 39 weeks), suggesting SFV co-infection may affect pathogenicity [58]. Similar co-infection studies in SFV-infected NPs have not been done.

## 6. SFV Cross-Species Transmission

Episodes of SFV transmission between NP species seems rare with only a few reported cases. A captive yellow-breasted capuchin (*Sapajus xanthosternos*) of the Cebidae family was found to be infected with an SFV that was phylogenetically more similar with an SFV from a spider monkey (*Ateles* species) of the Atelidae family [23]. Most likely, this cross-species transmission occurred while these monkeys were in captivity, since both primate species are not sympatric in Brazil. *Sapajus xanthosternos* are naturally found in the Caatinga forest of northeastern Brazil, while all species of *Ateles* inhabit the Amazon forest in northern Brazil. Phylogenetic analysis has also shown divergent SFV in *Leontopithecus* (tamarins) that do not cluster together, as would be expected under the co-evolution of SFV and host hypothesis, indicating at least one cross-species transmission in captivity [24]. Studies with free-living *Leontopithecus* will have to elucidate this finding.

Ancient cross-species and cross-genus SFV transmission may also have occurred in NPs and could explain the extant phylogenetic relations observed for SFVs in squirrel monkeys (*Saimiri* sp.) in the Cebidae family [14,23,25]. Squirrel monkey SFVs are more similar to those from monkeys in the Atelidae family (Figure 3) instead of the Cebine family (capuchins) as expected with a stable co-speciation history. Phylogenetic dating suggests that this cross-species transmission may have occurred at least 17 MYA with one or more SFV lineages not yet characterized, or possibly from the *Aotus* genus [25]. SFV cross-species transmissions have also been described for OWPs and appear to be equally infrequent [36,59]. 

To date, all persistent SFV infections detected in humans with documented viral nucleic acids originated by zoonotic transmissions of SFV infecting OWPs [60,61,62,63,64]. Cases of zoonotic transmission have been reported among individuals with exposure to nonhuman primates (NHPs), including veterinarians, keepers, biologists, researchers, and pet owners. Butchers and hunters can also be infected with SFV as OWP hunting and meat consumption is common in African villages and indigenous communities [65]. These workers and hunters have been infected with many different divergent OWP SFVs, including macaques, African green monkeys, chimpanzees, gorillas, mandrills, colobus monkeys, and various *Cercopithecus* species [63,66,67,68,69]. Interestingly, studies have shown that between 18 and 36% of individuals who were severely bitten or injured from hunting wild chimpanzees and gorillas in Cameroon and Gabon were SFV-positive [60,70], further supporting bites as the major transmission route. Longitudinal studies of persons infected with OWP SFVs have yet to show strong evidence of diseases associated with their zoonotic infections [57,58,59,60]. One recent case-control study reported potential hematological abnormalities in apparently healthy SFV-infected persons from Cameroon [66]. More systematic longitudinal studies are required to investigate SFV disease associations, including in persons co-infected with other retroviruses such as human immunodeficiency and human T-cell leukemia viruses. 

In contrast to OWPs, the potential for zoonotic transmission of NP SFVs to humans is less clear. A first study evaluated the presence of SFV among 116 primatologists, of which 69 were occupationally exposed to NPs [44]. While 12% (8/69) of the primatologists were positive for NP SFV by WB, no viral DNA was detected in the blood of these individuals, suggestive of exposure but not persistent infection or latent infection. Only four of the eight SFV-seropositive individuals reported accidents with NPs such as bites, scratches and injuries with contaminated sharp instruments. The other four workers reported contact with body fluids, but not parenterally, suggesting that contact with animal fluids without parenteral exposure may be sufficient for viral infection [44]. 

More recently, a study of persons occupationally exposed to SFV-infected NPs at a zoo and a primatology center in Brazil was reported. In this study, whole blood and oral swab samples were obtained from 56 individuals over three years (2011, 2012/2013 and 2014) [67]. A serological prevalence of 19% was found for NP anti-SFV antibodies, while—similar to the initial study described above—viral DNA was not detected in any of the sampled compartments. As issues related to the presence of PCR inhibitors in the DNA preparations were considered, the authors used different methods to clean the DNA from potential inhibitors, including the use of the OneStep PCR inhibitor removal kit (Zymo Research, Irvine, CA, USA) or of general column-based PCR purification kits after DNA extraction. However, even those measures failed to provide PCR-positive samples. Of the 12 SFV-seroreactive workers, 11 reported bites, scratches and/or direct contact with body fluids from NPs. Interestingly, for some of the workers more recently exposed to infected animals, a clearance of SFV antibodies was detected two years later compared to previous collection time points. WB-negative workers in this study reported contact with NPs for an average of 12 years, while the WB-positive workers reported NP contact for an average of only three years. 

Combined, these findings in persons exposed to NPs suggest possible control of SFV infection and antibody clearance, in contrast to human infection with OWP SFV. In these NP exposure cases, it is not known whether the virus has been truly eliminated from the body or if it is still present in another body compartment or reservoir. Additional follow-up testing of these workers and additional studies of persons exposed to NPs will help to elucidate the potential for zoonotic transmission of NP SFVs to humans. Exposure to NPs in Brazil can be frequent in forest parks and in large urban centers where primates have been observed feeding on household waste [68]. Evidence for disease in these workers with SFV-reactive serology results was not reported and clear evidence of disease associations has not been found in persons infected with OWP SFVs [64,68].

Like OWP SFVs, studies have documented that different human cells can be infected with NP SFVs, including strains of SFVaxx and SFVssc, but the cell tropism was distinct between strains. SFVaxx infected HT1080 (fibrosarcoma), MDA (mammary adenocarcinoma) and C33A (cervical carcinoma), but not AGS (adenocarcinoma), LN (lymphoblastoid) and LoVo (adenocarcinoma), while SFVssc infected only HT1080 [44]. Another study showed that the human TRIM5α did not affect the replication of SFVcja and SFVaxx [69], but restricted the replication of SFVssc [20]. These latter findings may help to explain the seroreactive but PCR-negative results in humans exposed to NP SFVs.

## 7. Perspectives

While considerable progress has been made in recent years to better understand the epidemiology and evolutionary history of NP SFVs, more research is needed. Additional SFV genomes should be sequenced, with emphasis on those infecting species from the Pitheciidae family (titi, saki and uacari monkeys) and the *Aotus* genus (owl monkeys), in order to clarify the evolutionary relationships of SFV among NPs, especially the unusual relationships of SFV that infect squirrel monkeys (*Saimiri* sp). Studies published to date with NPs have used a large number of specimens of various captive genera and species, but few specimens of each species. Future work should focus on studies of the viral epidemiology of wild NPs, with a reasonable number of specimens per species. RNA VL in oral tissues needs to be determined, as well as which tissues are targeted by SFV infection in NPs. Follow-up studies of workers with direct or indirect contact with NPs should continue to clarify whether SFV infection is resolved or if the virus persists in certain cells or body compartments. Additional studies of persons naturally exposed to NPs and their SFV infections are needed to define zoonotic risks for these viruses.

## Figures and Tables

**Table 1:** Simian foamy virus (SFV) diversity in neotropical primates revealed by virus detection and complete or partial genome characterization.

Primate Family	Genus	Common Name	Complete Genome ^1^	Partial Genome (LTR/*gag* and/or *pol*) ^1^	Diagnostic-PCR and/or qPCR and/or WB Serology ^1^
Cebidae	*Aotus*	owl monkey			SFVaaz, SFVatr, SFVani
	*Callimico*	marmoset			SFVcgo
	*Callithrix*	marmoset	SFVcja	SFVcge	SFVcau
	*Cebus*	capuchin		SFVcal	SFVcol
	*Leontopithecus*	tamarin		SFVlro, SFVlcm	SFVlcp
	*Mico*	marmoset			SFVmhu
	*Saguinus*	tamarin			SFVsbi, SFVsfu, SFVsmi, SFVsoe
	*Saimiri*	squirrel monkey	SFVssc	SFVsbo, SFVsus	
	*Sapajus*	capuchin	SFVsxa	SFVsap, SFVsfl, SFVsro	
Atelidae	*Alouatta*	howler monkey		SFVabe, SFVaca, SFVagu, SFVase	SFVapl
	*Ateles*	spider monkey	SFVaxx	SFVage, SFVahy, SFVach	SFVafu, SFVapn
	*Brachyteles*	wooly spider monkey	SFVbar		
	*Lagothrix*	wooly monkey		SFVlla	
Pitheciidae	*Cacajao*	uakari	___	SFVcca, SFVcme	
	*Callicebus*	titi	___	___	SFVcmo
	*Pithecia*	saki	___	SFVppi	

^1^ Species definition for SFV by primate genera: *Aloutta*: SFVabe (*Alouatta belzebul*), SFVaca (*Alouatta caraya*), SFVagu (*Alouatta guariba*), SFVase (*Alouatta seniculus*); SFVapl (*Alouatta palliata*); *Aotus*: SFVaaz (*Aotus azarae*), SFVatr (*Aotus trivirgatus*), SFVani (*Aotus nigriceps*); *Ateles*: SFVage (*Ateles geoffroyi*), SFVahy (*Ateles hybridus*), SFVach (*Ateles chamek*), SFVafu (*Ateles fusciceps*), SFVapn (*Ateles paniscus*), SFVaxx (*Ateles* sp.); *Brachyteles*: SFVbar (*Brachyteles aracnoides*); *Cacajao*: SFVcca (*Cacajao calvus*), SFVcme (*Cacajao melanocephalus*); *Callicebus*: SFVcmo (*Callicebus moloch*); *Callimico*: SFVcgo (*Callimico goeldii*); *Callithrix*: SFVcau (*Callithrix aurita*), SFVcge (*Callithrix geoffroyi*), SFVcja (*Callithrix jaccus*); *Cebus:* SFVcal (*Cebus albifrons*), SFVcol (*Cebus olivaceus*); *Lagothrix*: SFVlia (*Lagothrix lagotricha*) *Leontopithecus:* SFVlcm (*Leontopithecus chrysomelas*), SFVlcp (*Leontopithecus chrysopygus*), SFVlro (*Leontopithecus rosalia*); *Mico*: SFVmhu (*Mico humeralifer*); *Pithecia*: SFVppi (*Pithecia pithecia*); *Saguinus*: SFVsbi (*Saguinus bicolor*), SFVsfu (*Saguinus fuscicollis*), SFVsmi (*Saguinus midas*), SFVsoe (*Saguinus oedipus*); *Saimiri*: SFVsbo (*Saimiri boliviensis*), SFVssc (*Saimiri sciureus*), SFVsbo (*Saimiri ustus*); *Sapajus*: SFVsap (*Sapajus apella*), SFVsfl (*Sapajus flavius*), SFVsro (*Sapajus robustus*), SFVsxa (*Sapajus xanthosternos*).

**Table 2:** Simian foamy virus prevalence in neotropical primates.

Study	Methodology	Sites	Prevalence
Hooks, 1975 [2]	Serology	Colony	61%
Marczynska et al., 1981 [18]	Serology	Colony	54%
Muniz et al., 2013 [23]	Diag. PCR ^1^	Brazilian zoo and primatology center Wild primates	23% 29%
Ghersi et al., 2015 [25]	Serology and Diag. PCR	Peruvian and US zoos Peruvian rescue center Illegal trade market	45–47% 19%
Muniz et al., 2015 [24]	Serology and Diag. PCR	Brazilian zoo and primatology center	51%

^1^ Diagnostic PCR.
